# Comparison of Outcomes Between Milrinone and Dobutamine in Patients With Cardiogenic Shock: A Meta-Analysis of Randomized Control Trials and Observational Studies

**DOI:** 10.7759/cureus.54948

**Published:** 2024-02-26

**Authors:** Chenyue Fan, Calvin R Wei, Neelum Ali

**Affiliations:** 1 Research and Development, Shing Huei Group, Taipei, TWN; 2 Internal Medicine, University of Health Sciences, Lahore, PAK

**Keywords:** outcomes, meta-analysis, cardiogenic shock, dobutamine, milrinone

## Abstract

The aim of this systematic review and meta-analysis was to compare the outcomes between milrinone and dobutamine in patients with cardiogenic shock. The search strategy involved a comprehensive exploration of electronic databases, including PubMed, EMBASE, Cochrane Library, and Scopus from the the inception of each database up to the 31st of January 2024. A combination of keywords and Medical Subject Headings (MeSH) terms was employed to identify relevant studies. The outcomes assessed in this meta-analysis included all-cause in-hospital mortality, length of intensive care unit (ICU stay), and length of hospital stay. A total of seven studies were included in this meta-analysis enrolling 3,841 patients (2,331 in the dobutamine group and 1,510 in the milrinone group). Pooled analysis showed that the risk of all-cause mortality was significantly higher in patients receiving dobutamine compared to patients receiving milrinone (relative risk (RR): 1.43, 95% confidence interval (CI): 1.02 to 2.01, p-value: 0.04). However, the length of hospital stay and length of ICU stay were not significantly different between the two groups. Limited data are available to favor the use of one inotropic agent over another. Dobutamine might lead to a shorter hospital length of stay, but there is also a risk of increased all-cause mortality. Larger randomized studies with adequate power are needed to validate these observations.

## Introduction and background

Cardiogenic shock is delineated as a condition characterized by diminished cardiac output, leading to discernible clinical and biochemical signs of inadequate organ perfusion [1-2]. It is marked by a deterioration in cardiac function culminating in a notable reduction in the cardiac output and an insufficiently effective circulating blood volume, precipitating acute peripheral circulatory failure of severe magnitude. The mortality rate associated with cardiogenic shock varies widely, ranging from 50% to 80% [3]. The primary cause of cardiogenic shock is typically acute myocardial infarction (AMI), which accounts for approximately 80% of cases [4]. Despite notable strides in available mechanical circulatory support, studies conducted thus far have not yielded significant improvements in clinical outcomes. Emergency revascularization stands out as the sole therapy demonstrated to mitigate the risk of mortality among patients with myocardial infarction complicated by cardiogenic shock [5-6].

The etiology of cardiogenic shock often stems from various cardiac pathologies, including AMI, severe valvular disease, myocarditis, or decompensated heart failure. While AMI remains the predominant cause, it is essential to recognize and address the underlying cardiac dysfunction or structural abnormalities contributing to the development of cardiogenic shock. In addition, the severity and extent of myocardial damage, as well as the presence of comorbidities, play crucial roles in determining the overall prognosis and management approach [1-2]. The objective of cardiogenic shock treatment can be divided into two primary categories: first, to address and identify the underlying cause, and second, to furnish adequate hemodynamic support through the administration of vasoactive medications. Vasopressors or inotropes have assumed a pivotal role in hemodynamic management for the majority of cardiogenic shock patients owing to their capacity to diminish adrenergic stress (decathecolaminization) [7]. These medications are commonly employed as adjunctive therapy until definitive treatments can be implemented. However, despite their widespread use, vasopressors or inotropes may elevate left ventricular afterload, leading to increased myocardial oxygen demand, larger infarct size, and a heightened risk of arrhythmia [8]. Furthermore, the dosing regimen for vasoactive medications necessitates meticulous titration to achieve optimal hemodynamic support while minimizing adverse effects. Close monitoring of vital signs, cardiac output, and end-organ perfusion is imperative to guide therapeutic adjustments and ensure therapeutic efficacy [6].

Norepinephrine emerges as the preferred vasopressor in cases of cardiogenic shock due to its association with fewer arrhythmic complications and reduced mortality compared to dopamine. Nevertheless, observational evidence hints at the potential for enhanced survival with the adjunctive use of an inodilator, which not only acts as a positive inotrope but also reduces afterload [9-10]. Among the commonly prescribed inodilators, milrinone and dobutamine stand out; however, their distinct pharmacodynamic and pharmacokinetic profiles may yield differing outcomes [11]. While various studies have examined the safety and efficacy of milrinone versus dobutamine, most have adopted an observational approach. Since the most recent meta-analysis [12], there have been relatively few investigations specifically focusing on the efficacy and safety of these agents in cardiogenic shock. Thus, the present systematic review and meta-analysis aim to fill this gap by incorporating recently conducted studies, aiming to provide an updated comparison of the outcomes between milrinone and dobutamine in patients experiencing cardiogenic shock.

## Review

Methodology

Search Strategy 

The search strategy involved a comprehensive exploration of electronic databases, including PubMed, EMBASE, Cochrane Library, and Scopus. A combination of keywords and Medical Subject Headings (MeSH) terms was employed to identify relevant studies. Keywords used to search for relevant articles included “milrinone,” “dobutamine,” and “cardiogenic shock.” The search was conducted from the inception of each database up to the 31st of January 2024. In addition, manual searches of pertinent journals, conference proceedings, and gray literature supplemented the electronic searches. Reference lists of all included articles were manually searched to identify relevant studies. A search was performed by two authors independently, and any disagreement between two authors was resolved through discussion.

Study Selection 

Study selection adhered to predefined inclusion criteria. The population of interest included adult patients diagnosed with cardiogenic shock. The interventions under consideration were the administration of milrinone and dobutamine. Only studies employing randomized controlled trials (RCTs) and observational designs were included. Two reviewers independently carried out the selection process. All studies were imported into EndNote X9 version (Clarivate, United Kingdom). After removing duplicates, articles were screened using abstracts and titles. Studies passed through the initial screening process were detailed screened by the same two authors based on predefined inclusion and exclusion criteria. Disagreement between two authors was resolved through consensus or consultation with a third reviewer if necessary. 

*Data Extraction, Outcomes, and Quality Assessment* 

A systematic approach was employed for data extraction from selected studies. A standardized data extraction form was developed on Microsoft Excel (Microsoft Corporation, USA) and used consistently across all included studies. Data extracted from the included studies were study characteristics (author, publication year, study design, country, and sample size), participant characteristics (mean age, gender, diabetes, and hypertension), and outcomes of interest (mortality rate). Data extraction was performed by two authors independently (CF and CW), and any disagreement between two authors was resolved through discussion. Quality assessment was conducted using established tools tailored to the study design. The Cochrane risk-of-bias tool was applied for RCTs, while the Newcastle-Ottawa Scale was utilized for observational studies. Two independent reviewers (CF and CW) assessed the quality of each included study, and any disagreements were resolved through discussion or consultation with a third reviewer (NA).

Data Analysis

The data analysis was conducted using RevMan 5.4.1 (The Cochrane Collaboration, Oxford, United Kingdom). Categorical outcomes were presented as risk ratios (RRs) with corresponding 95% confidence intervals (CI), while continuous variables were expressed as mean differences (MDs) with 95% CI. Significance was determined by a p-value less than 0.05. A random-effects meta-analysis was employed to evaluate efficacy, accompanied by an assessment of statistical heterogeneity using the I² statistic. Significant heterogeneity was indicated by an I² value exceeding 50%. In addition, a sensitivity analysis was conducted by iteratively removing one study at a time.

Results

**Figure 1:**
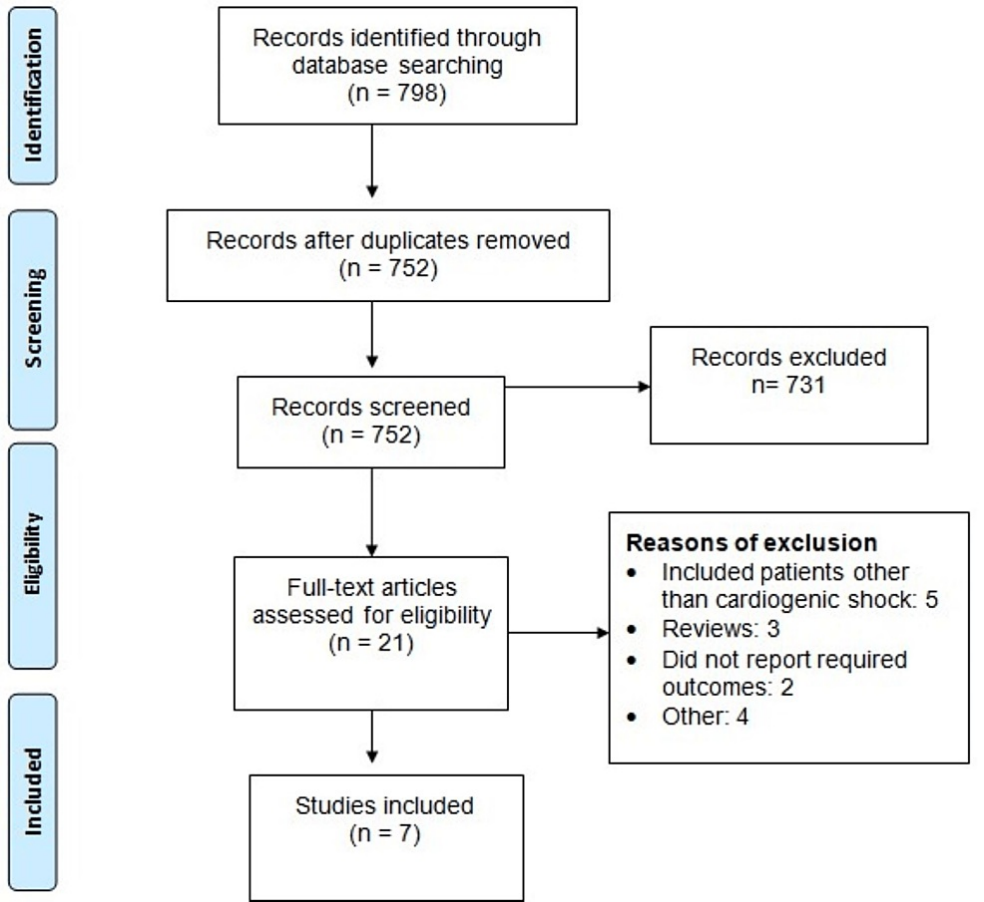
Preferred Reporting Items for Systematic Reviews and Meta-Analyses (PRISMA) flowchart of study selection

We identified 798 records. Using inclusion and exclusion criteria, we initially evaluated the abstract and titles followed by full-text screening of eligible studies. Finally, seven studies were included in this meta-analysis, describing 3,841 patients (2,331 in the dobutamine group and 1,510 in the milrinone group). Figure 1 shows the process of study selection. 

Characteristics of the Included Studies

Table 1 shows the characteristics of the included studies. Among seven studies, six were observational and one was RCT focused on cardiogenic shock patients. Four studies were performed in the United States, while one each was conducted in China, the Netherlands, and Canada. Table 2 presents the quality assessment of all the included studies. 

**Table 1:** Characteristics of the included studies NR: not reported; MI: myocardial infarction; RCT: randomized control trial

Author	Region	Study design	Groups	Sample size	Age (mean)	Male (n)	Hypertension (n)	Diabetes (n)	History of MI
Berg et al., 2023 [13]	Netherlands	Observational	Dobutamine	503	NR	NR	NR	NR	NR
			Milrinone	267					
Gao et al., 2021 [14]	United States	Observational	Dobutamine	558	67.31	344	289	184	111
			Milrinone	261	64.82	163	139	86	62
Lewis et al., 2019 [15]	United States	Observational	Dobutamine	50	75	23	38	16	10
			Milrinone	50	72.5	28	31	12	11
Mathew et al., 2021 [16]	United States	RCT	Dobutamine	96	72	62	NR	NR	29
			Milrinone	96	68.9	60			39
Nandkeoiyar et al., 2021 [17]	United States	Observational	Dobutamine	256	NR	NR	NR	NR	NR
			Milrinone	70					
Rodenas-Alesina et al., 2023 [18]	Canada	Observational	Dobutamine	207	63	147	88	63	42
			Milrinone	366	60	259	148	104	77
Sasmita et al., 2023 [19]	China	Observational	Dobutamine	661	NR	NR	NR	NR	NR
			Milrinone	400					

**Table 2:** Quality assessment of the included studies

Quality assessment for observational studies					
Study ID	Selection	Comparability	Outcome	Overall	
Berg et al., 2023 [13]	2	1	2	Fair	
Gao et al., 2021 [14]	3	2	2	Good	
Lewis et al., 2019 [15]	3	2	3	Fair	
Nandkeoiyar et al., 2021 [17]	4	2	2	Good	
Rodenas-Alesina et al., 2023 [18]	4	2	3	Good	
Sasmita et al., 2023 [19]	2	2	2	Fair	
Quality assessment for randomized controlled trials					
Study ID	Selection	Performance	Attrition	Reporting	Other
Mathew et al., 2021 [16]	Low	Low	Low	Unclear	Low

All-Cause Mortality 

**Figure 2:**
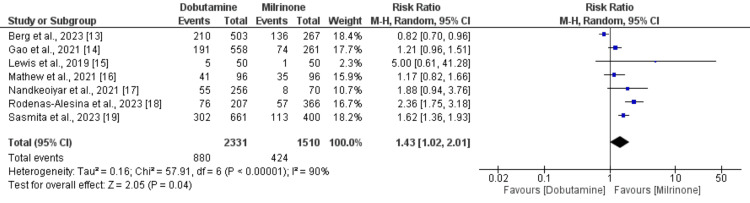
Comparison of all-cause mortality between dobutamine and milrinone Sources: References [13-19]

Seven studies were included in the pooled analysis of comparing the all-cause mortality between the dobutamine and milrinone groups. As shown in Figure 2, the risk of all-cause mortality was significantly higher in patients receiving dobutamine compared to patients receiving milrinone (RR: 1.43, 95% CI: 1.02 to 2.01, p-value: 0.04). High heterogeneity was reported among the study results (I-square: 95%). We performed sensitivity analysis by removing one study at a time, and the results are shown in Table 3. After removing one study at a time, we still found a higher risk of all-cause mortality in patients receiving dobutamine. 

**Table 3:** Sensitivity analysis RR: risk ratio; CI: confidence interval

Author	RR (95% CI)	I-square
Berg et al., 2023 [13]	1.58 (1.23 to 2.04)	70%
Gao et al., 2021 [14]	1.50 (0.98 to 2.31)	91%
Lewis et al., 2019 [15]	1.39 (0.98 to 1.96)	91%
Mathew et al.,2021 [16]	1.49 (1.01 to 2.02)	91%
Nandkeoiyar et al., 2021 [17]	1.38 (0.96 to 1.99)	91%
Rodenas-Alesina et al., 2023 [18]	1.27 (0.92 to 1.77)	87%
Sasmita et al., 2023 [19]	1.40 (0.93 to 2.11)	89%

Hospital Length of Stay (Days) 

**Figure 3:**
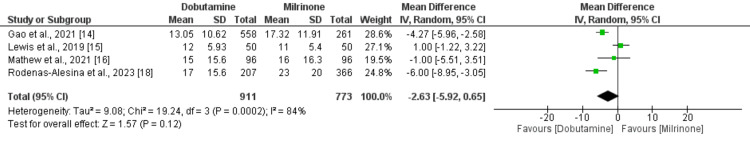
Comparison of hospital stay duration between dobutamine and milrinone Sources: References [14-16, 18]

Four studies were included in the pooled analysis comparing the length of hospital stay between the dobutamine and milrinone groups, and the results are shown in Figure 3. Pooled results showed that the length of hospital stay was not significantly different between the two groups (mean difference (MD): -1.99, 95% CI: -6.70 to 2.72, p-value: 0.41). High heterogeneity was reported among the study results (I-square: 86%). 

ICU Length of Stay (Days)

**Figure 4:**
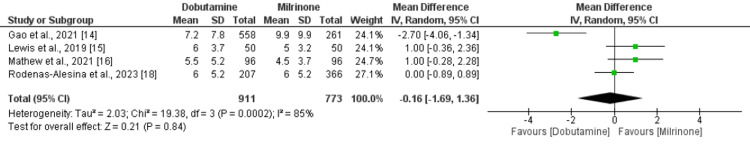
Comparison of ICU stay between dobutamine and milrinone Sources: References [14-16,18]

The pooled analysis encompassed four studies investigating the comparison of ICU stay duration between the dobutamine and milrinone treatment groups. The pooled findings, depicted in Figure 4, indicated no statistically significant difference in the length of ICU stay between the two groups (MD: -0.16, 95% CI: -1.69 to 1.36, p-value: 0.84). Notably, a high level of heterogeneity was observed among the study results, with an I-square value of 85%.

Discussion

In this systematic review and meta-analysis, we investigated the comparative effectiveness of dobutamine and milrinone in patients diagnosed with cardiogenic shock. Our analysis revealed a higher risk of all-cause mortality among dobutamine-treated patients compared to those administered milrinone. However, no significant differences were observed between the two groups concerning the length of stay in the intensive care unit (ICU) or the duration of hospitalization. These findings align with previous systematic reviews and meta-analyses conducted by Abdel-Razek et al. [12]. However, based on the available evidence, we refrain from making strong recommendations regarding the preferential use of one inotrope over the other in hospitalized patients. Nonetheless, our results may indicate the need for larger studies focusing specifically on mortality outcomes to provide more definitive conclusions.

Data guiding the selection of inotropic agents for patients with cardiogenic shock are scarce. The Sepsis Occurrence in Acutely Ill Patients II (SOAP II) trial, which compared dopamine with norepinephrine in shock management, including a subset of 280 patients with cardiogenic shock, found no significant difference in mortality between the treatment modalities [20]. Similarly, previous comparisons of milrinone and dobutamine, such as a single randomized trial involving 36 hospitalized patients awaiting cardiac transplantation, demonstrated no disparity in in-hospital mortality [21]. Most observational data lean toward a reduction in all-cause in-hospital mortality associated with milrinone. While this trend may suggest potential benefits, the absence of robust data complicates drawing definitive conclusions. Conversely, RCTs focusing exclusively on cardiogenic shock patients have reported a 20% relative reduction in mortality with milrinone; however, the difference was not statistically significant [16]. Therefore, while inotropic agents constitute a cornerstone of therapy for cardiogenic shock, our meta-analysis addresses a critical gap in understanding the management of this condition.

In terms of ICU length of stay, the mean duration of hospitalization did not exhibit significant differences between the two groups. However, only one of the included studies, which was observational in nature, favored dobutamine [14]. Other studies included in the analysis did not indicate any notable disparities between the two groups regarding ICU length of stay [15-16,18]. This discrepancy might reflect the patient demographics targeted by each inotropic agent in the observational studies. Milrinone has been predominantly utilized as a long-term therapy for patients with end-stage heart failure, leading to its more frequent administration in patients necessitating prolonged hospital admissions for comprehensive heart failure assessments [22].

Emerging evidence suggests that milrinone, a phosphodiesterase inhibitor, may offer particular benefits for patients with cardiogenic shock complicated by pulmonary hypertension or RV failure. Unlike dobutamine, milrinone exerts its positive inotropic effects without significantly increasing myocardial oxygen demand, making it a potentially favorable option in these critical patients [22]. In addition, milrinone's pulmonary vasodilatory properties may help alleviate pulmonary hypertension and reduce RV afterload, thereby improving cardiac output and hemodynamic stability [21]. Moreover, despite its higher cost compared to dobutamine, milrinone is more commonly prescribed to patients eligible for advanced support, including heart transplantation [21]. It is plausible that dobutamine may contribute to early mortality among patients, thereby resulting in shorter hospital stays, or conversely, it may prompt swifter recovery. Nonetheless, these observations necessitate confirmation through larger prospective trials [12].

Our analysis encompasses both RCTs and observational data, primarily due to the scarcity of robust studies investigating the efficacy of dobutamine and milrinone in treating cardiogenic shock. The inclination toward reduced mortality associated with milrinone, as observed in RCTs, is further reinforced by similar trends evident across all available studies addressing this subject. This consistent pattern observed across both observational and randomized data underscores the necessity for further inquiry through a well-powered study to thoroughly explore the potential mortality advantages associated with milrinone compared to dobutamine among patients suffering from cardiogenic shock.

Our study is subject to several limitations. First, the majority of randomized data originate from a single randomized controlled trial (14), while the remaining data are derived from observational studies. Consequently, inherent biases present in observational studies may confound the results and hinder our ability to make definitive recommendations regarding the choice of inotrope. Second, the absence of long-term outcome data in the included studies prevents us from drawing conclusions regarding long-term outcomes. In addition, considerable heterogeneity exists among the included patients, with some studies focusing solely on postcardiac surgery patients or those awaiting transplantation, while others encompass a broader spectrum of patients with cardiogenic shock, irrespective of etiology. Heterogeneity is also due to differences in the study population and study setting. Lastly, we were not able to perform subgroup analysis due to the lack of individual-level data.

## Conclusions

We conducted this review to compare the outcomes between dobutamine and milrinone in patients with cardiogenic shock. Overall, seven studies were included in this meta-analysis, and the pooled analysis has reported the heightened risk of all-cause mortality associated with dobutamine compared to milrinone in patients with cardiogenic shock. However, no significant differences were found in ICU or hospital stay durations between the two groups. While our findings align with previous analyses, larger prospective trials are needed to validate these results and explore the potential benefits of milrinone over dobutamine in this population.
